# Engineering the Active Site Pocket to Enhance the Catalytic Efficiency of a Novel Feruloyl Esterase Derived From Human Intestinal Bacteria *Dorea formicigenerans*

**DOI:** 10.3389/fbioe.2022.936914

**Published:** 2022-06-20

**Authors:** Yang Shen, Yulu Wang, Xue Wei, Boting Wen, Shujun Liu, Huishuang Tan, Jingjian Zhang, Shuli Shao, Fengjiao Xin

**Affiliations:** ^1^ Department of Life Science and Agroforestry, Qiqihar University, Qiqihar, China; ^2^ Laboratory of Biomanufacturing and Food Engineering, Institute of Food Science and Technology, Chinese Academy of Agricultural Sciences, Beijing, China; ^3^ Key Laboratory of Ministry of Education for Protein Science, School of Life Sciences, Tsinghua University, Beijing, China; ^4^ Cangzhou Academy of Agriculture and Forestry Sciences, Cangzhou, China

**Keywords:** feruloyl esterase, gut microbiota, Dorea formicigenerans, AlphaFold2, rational design, substrate access tunnel

## Abstract

The human gut microbiota play essential roles in metabolism and human health, especially by enzymatically utilizing dietary fiber that the host cannot directly digest and releasing functional components including short-chain fatty acids (SCFAs) and hydroxycinnamic acids (e.g., ferulic acid). In our previous study, seven potential feruloyl esterase (FAE) genes were identified from the gut microbiota. In the current work, one of the genes encoding a novel FAE (*Df*FAE) from *Dorea formicigenerans* of *Firmicutes* was bacterially expressed, purified and characterized. The 30.5 kDa type-A *Df*FAE has an optimum pH and temperature of 8.4 and 40 °C, respectively, exhibiting a higher substrate specificity toward short-chain acyl-ester substrate (*p*NPA). The AlphaFold2 based *ab initio* structural modeling revealed a five α-helices cap domain that shaped an unusually narrow and deep active site pocket containing a specific substrate access tunnel in *Df*FAE. Furthermore, rational design strategy was subjected to the active site pocket in an aim of improving its enzymatic activities. The mutants V252A, N156A, W255A, P149A, and P186A showed 1.8 to 5.7-fold increase in catalytic efficiency toward *p*NPA, while W255A also exhibited altered substrate preference toward long-chain substrate *p*NPO (45.5-fold). This study highlighted an unusual active site architecture in *Df*FAE that influenced its substrate selectivity and illustrated the applicability of rational design for enhanced enzymatic properties.

## Introduction

The concept of dietary fiber and prebiotics has long been strongly promoted by the nutritional community. These dietary components resist breakdown in the human small intestine and are utilized by gut microbes in the colon that live in symbiosis with the body (Slavin, 2013). The activities of gut microbiota profoundly influence the metabolism and human health (Eckburg et al., 2005; Sonnenburg et al., 2010). Xylan, the second most abundant saccharides in plant kingdom, is an important constituent of dietary fiber and prebiotic supplements. Structurally, the backbone of xylan commonly consists of β-1,4-linked D-xylopyranose residues that are often substituted with a variety of side chains, including glucuronyl groups, acetyl groups and arabinosyl groups. Ferulic acid or other hydroxycinnamic acids, are additionally estified to the C-5 position of some arabinosyl moieties, which may further crosslink to lignin or neighboring xylan chains by forming diferulate, thus increasing the recalcitrance of plant saccharides to enzymatic hydrolysis in the colon of humans (Scheller and Ulvskov, 2010).

Ferulic acid or feruloyl esterases (FAEs, EC 3.1.1.73) are a subgroup of carboxylic ester hydrolases, which catalyze the cleavage of the ester bond between arabinofuranose and hydroxycinnamic acid. Removing the cross-linked ferulic acid side chain is necessary for the efficient degradation of feruloylated xylan polymers (Williamson et al., 1998; Faulds, 2010; Scheller and Ulvskov, 2010). Hence, FAEs possess wide biotechnological application in the biofuel, food and pharmaceutical industries (Oliveira et al., 2019). FAEs belong to Carbohydrate Esterase Family 1 (CE1) and initially categorized into four subclasses (A, B, C and D) according to their substrate specificity and sequence similarity. In general, FAEs display a common catalytic mechanism involving the Ser-His-Asp catalytic triad, and adopt the structure of canonical α/β hydrolase fold, which are usually composed of the core domain (also known as the catalytic domain) containing the catalytic triad and the cap domain positioning atop the core domain (Oliveira et al., 2019). The cap domain confines the active site cavity and its conformation influences substrate recognition and catalytic properties in different FAEs (Bauer et al., 2020).

FAEs are mostly found in microorganisms, but also in edible mushrooms (Wang et al., 2014) and plants (de O Buanafina et al., 2019). FAEs of fungal origin, such as *Aspergillus niger* (Hermoso et al., 2004), have been extensively studied. Recently, FAEs of gut symbiotic bacteria have gained increasing attention. An increasing number of FAEs have been identified from *Bifidobacteria* (Raimondi et al., 2015; Fritsch et al., 2017), *Lactobacillus* (Lai et al., 2009), or *Bacteroides* (Wefers et al., 2017), but they display distinct catalytic features with respect to properties such as substrate specificities and optimal reaction conditions. These gut microbial-derived FAEs are the main pathway for the release of hydroxycinnamic acids from dietary fiber, which are released in the form of free acids and absorbed into the circulatory system for action (Andreasen et al., 2001; Faulds, 2010). Hydroxycinnamic acids play a positive role in human health due to their excellent antioxidant, anti-inflammatory, anti-diabetes, anti-cancer and neuroprotective capacities (El-Seedi et al., 2012). Thus, the discovery and characterization of novel gut-derived FAEs is of great importance to explain the intestinal hydrolysis and releasing mechanism of dietary hydroxycinnamic acids on behalf of the human health.

In our previous work, seven potential FAE genes were identified from *in vitro* fermentation of human fecal slurry using metagenomic sequencing (Chen et al., 2020) in which one FAE from *Alistipes shahii* (*As*FAE) was characterized (Wei et al., 2021). Here, a novel FAE from *Dorea formicigenerans* (*Df*FAE) of *Firmicutes* was bacterially expressed and purified to assess its enzymatical properties. *Df*FAE belongs to type-A FAE and has a higher preference for hydrolyzing short-chain ester substrate (*p*-nitrophenyl acetate, *p*NPA), which shows superior catalytic activity than *As*FAE. To understand the structural basis of *Df*FAE catalytic properties, an *ab initio* modeling using AlphaFold2 was generated, which highlighted a relatively narrow and deep substrate binding pocket including a specific substrate access tunnel. Then site-specific mutagenesis and kinetic studies were carried out on residues around the active site tunnel, which identified five mutants with improved catalytic efficiency and/or broadened substrate preference. *In silico* analysis shed light on the possible molecular mechanisms underlying improved enzymatic properties. Collectively, this study presented the biochemical, structural and rational design studies of a novel FAE derived from *Dorea formicigenerans*, sharpening our mechanistic understanding of diverse mode of action in various FAEs.

## Materials and Methods

### Reagents

The vector pET-28a (+) for plasmid construction was obtained from Novagen (Madison, WI, United States). The *Escherichia coli* (*E. coli*) *Trans*1 (T1) strain purchased from TransGen Biotech (Beijing, China) was used to recombinant plasmids amplification, and the *E. coli* T7 *Express* strain purchased from Biomed (Beijing, China) was used to recombinant proteins expression.

A series of substrates used in this experiment: *p*-nitrophenyl acetate (*p*NPA), *p*-nitrophenyl butyrate (*p*NPB), and *p*-nitrophenyl octanoate (*p*NPO) were purchased from Sigma-Aldrich (St. Louis, MO, United States), and *p*-nitrophenyl trans-ferulate (*p*NPF) was purchased from MREDA (Columbia, MD, United States); methyl ferulate (MFA), methyl sinapinate (MSA), methyl caffeate (MCA) and methyl *p*-coumarate (M*p*CA) were purchased from TCI (Shanghai, China); the standards of ferulic acid (FA), sinapic acid (SA), caffeic acid (CA) and *p*-coumaric acid (*p*CA) were purchased from Sigma-Aldrich (St. Louis, MO, United States).

### Sequence Alignment and Phylogenetic Tree Analysis

The sequence similarity searches were carried out using the Blastp search engine from the National Center for Biotechnology Information (NCBI) database in conjunction with the PDB database. The SingnalP 5.0 was applied to predict the sequence of potential signal peptides. ClustalX version 2.0 (Larkin et al., 2007) and ESPript version 3.0 (Robert and Gouet, 2014) were used to align multiple sequences. The phylogenetic tree was built with the maximum likelihood estimation by MEGA version 11.0 (Tamura et al., 2021).

### Gene Cloning, Mutation, Heterologous Expression and Purification

The accession number of *Df*fae sequence is WP_117657856.1 in the NCBI database. The cDNA sequence after *E. coli* preference codon optimization was synthesized (BGI-write, Beijing, China), and restriction endonuclease sites, *Bam*H I and *Xho* I (Thermo Fisher Scientific, Hudson, NH, United States), were added at both ends of the sequence. Then the target fragment was inserted into the restriction enzyme-digested pET-28a (+) plasmid and transmitted into *E. coli* T7 for expression.

The mutagenesis were carried out standard Quikchange PCR procedure. pET28a-*Df*fae was used as the template and specific primers were used for site-directed mutagenesis. The original template was digested by *Dpn* I enzyme (Thermo Fisher Scientific, Hudson, NH, United States) and then transformed into *E. coli* T1 for amplification. Clones successfully mutated were verified by DNA sequencing.

*E. coli* T7 cells containing *Df*fae and its mutants were cultured in Luria-Bertani (LB) medium adding 100 μg ml^−1^ kanamycin at 37 °C, and shaked at 220 rpm for 3–4 h. When OD_600_ came to 0.8, expression of protein was induced by 0.2 mM isopropyl thiogalactoside (IPTG) at 16°C for 12 h. The cultures were centrifuged at 5,000 rpm and resuspended in 50 mM Tris-HCl (pH 8.0) buffer supplemented with 300 mM NaCl, 10 mM MgCl_2_, 1 mM phenylmethanesulfonyl fluoride (PMSF), 5 mg L^−1^ lysozyme, 0.03 mg L^−1^ DNase I. High pressure homogenization was used to lyse the cells, and the cell fragment supernatant was loaded on affinity chromatography (Ni^2+^-NTA, Qiagen, Hilden, Germany) eluted with 300 mM imidazole. The proteins were further purified using anion exchange column chromatography (Source 15Q, GE Healthcare Life Sciences, Issaquah, WA, United States) and gel filtration chromatography (Superdex™ 200 Increase 10/300 GL column, GE Healthcare Life Sciences, Issaquah, WA, United States). Ultimately, the purified *Df*FAE was analyzed by 12% SDS-PAGE and quantified by Bradford protein quantification kit (Solarbio, Beijing, China), and then stored at -80 °C for later use.

### Analysis of Enzymatic Properties

The basic biochemical properties of *Df*FAE were measured using a spectrophotometer (SpectraMax 190, Molecular Devices) with *p*NPA (dissolved in DMSO) as the substrate. The reactant mixture (total volume was 0.3 ml) included 0.3 mM *p*NPA and 0.3 μM *Df*FAE, and 2.5% Ttrion X-100 was added to all buffer solutions to stabilize the substrate in these experiments. The pH optima was determined at 40°C by reacting in 10 mM different buffers, including phosphate-citrate buffer (pH 4.0 and 5.0), K_2_HPO_4_-KH_2_PO_4_ buffer (pH 6.0, 6.6 and 7.0) and Tris-HCl buffer (pH 7.5, 8.0, 8.4 and 8.9). For pH stability measurements, the residual activity was detected at 40°C and pH 8.4, after incubating *Df*FAE in different pH buffers (pH 4.0–8.9) at 4°C for 4 h. The temperature optima was determined at different temperatures of 4 °C and 20–60°C (10°C intervals) in 10 mM Tris-HCl buffer (pH 8.4). The temperature stability was assayed by incubating *Df*FAE in the optimum pH buffer at different temperatures of 4°C and 20–60°C (10°C intervals) for 0.25, 0.75, 1, 2 and 4 h, and the residual activity was detected under the optimum pH and temperature conditions. To investigate the effects of metal ions and chemicals, *Df*FAE was incubated in 10 mM Tris-HCl buffer (pH 8.4) containing different metal ions and detergents at a final concentration of 1 mM (w/v) and 1% (v/v), respectively, for 15 min at room temperature. Then the residual activity was measured under the optimum pH and temperature conditions. The enzymatic activity without additives was defined as 100%. Three replicate experiments were performed for each reaction, and all data were shown as averages with the standard deviation.

### Kinetic Parameters of *Df*FAE and Its Mutants

The kinetic properties of *Df*FAE and its mutants were assayed in 10 mM Tris-HCl buffer (pH 8.4) containing 2.5% Triton X-100 at 40°C, using different concentrations (0.25–2 mM) of *p*NPA, *p*NPB and *p*NPO as substrates. The amount of produced *p*NP was detected at 405 nm and calculated based on the *p*NP standard curve. The initial rate of *p*NP release was plotted against substrate concentration, and the Michaelis-Menten constant (*K*_m_) and maximum reaction velocity (*V*_max_) were calculated by nonlinear regression analysis using Graphpad Prism eight software. The turnover number (*k*_cat_) was calculated as *V*_max_ divided by the corresponding enzymatic concentration.

### High Performance Liquid Chromatography (HPLC) Analysis

MFA, MSA, MCA and M*p*CA were used as substrates to verify the feruloyl esterase activity of *Df*FAE. The reaction mixture contained 1.25 mM substrate, 10 mM Tris-HCl (pH 8.4) and 2.5% Triton X-100. The reaction was initiated by adding 8 μM *Df*FAE at 40°C for 1 h, followed by the addition of equal volume of methanol to terminate the reaction. After being filtered through an organic membranefilter with a pore size of 0.22 μm, the samples were loaded onto the column and HPLC analysis was performed. The Agilent ZORBAX SB-C18 (4.6 × 250 mm, 5 μm) column was used with mobile phase A (water-formic acid, 99:1, v/v) and mobile phase B (methanol-formic acid, 99:1, v/v). The elution procedure was as follows: the total time was set to 25 min, at a flow rate of 0.5 ml/min, 0–9.5 min, 40% mobile phase B; 9.5–19 min, 85% mobile phase B; and 19–25 min returned to 40% mobile phase B. The column was finally equilibrated by the first-step conditions of the elution procedure. The product profiles were monitored at 323 nm and identified by comparing their retention periods to the standards’ retention times.

### Structural Prediction and Molecular Docking

The structural model of *Df*FAE was constructed using the open-source program AlphaFold2 (https://github.com/deepmind/alphafold). The program returned a total of five results, which were ranked as rank0-4 in descending order of accuracy, and the best prediction rank0 was selected for the following analysis. 98% of the residues under this model had pLDDT (predicted Local Distance Difference Test) scores greater than 80, hence results were generally reliable. Molecular dockings were carried out using the open-source program AutoDock_Vina (https://vina.scripps.edu/). The ligands (*p*NPA, *p*NPB and *p*NPO) structures were downloaded from NCBI PubChem Database (https://pubchem.ncbi.nlm.nih.gov/). The ligand-free pdb files of *Df*FAE and ligands were converted into pdbqt files using MGLTools (http://mgltools.scripps.edu/), a grid box of 50 × 50 × 50 Å^3^ was generated to cover the active site. The dock program with default parameters generated the top 20 best affinity results for each ligand, and the reasonable conformation with the lowest binding energy was selected for subsequent analysis. The structural comparison, visualization and homology modeling of W255A mutant were performed with PyMOL 2.3 software.

## Results and Discussion

### Sequence Analysis and Identification of *Df*FAE as a Feruloyl Esterase

**FIGURE 1:**
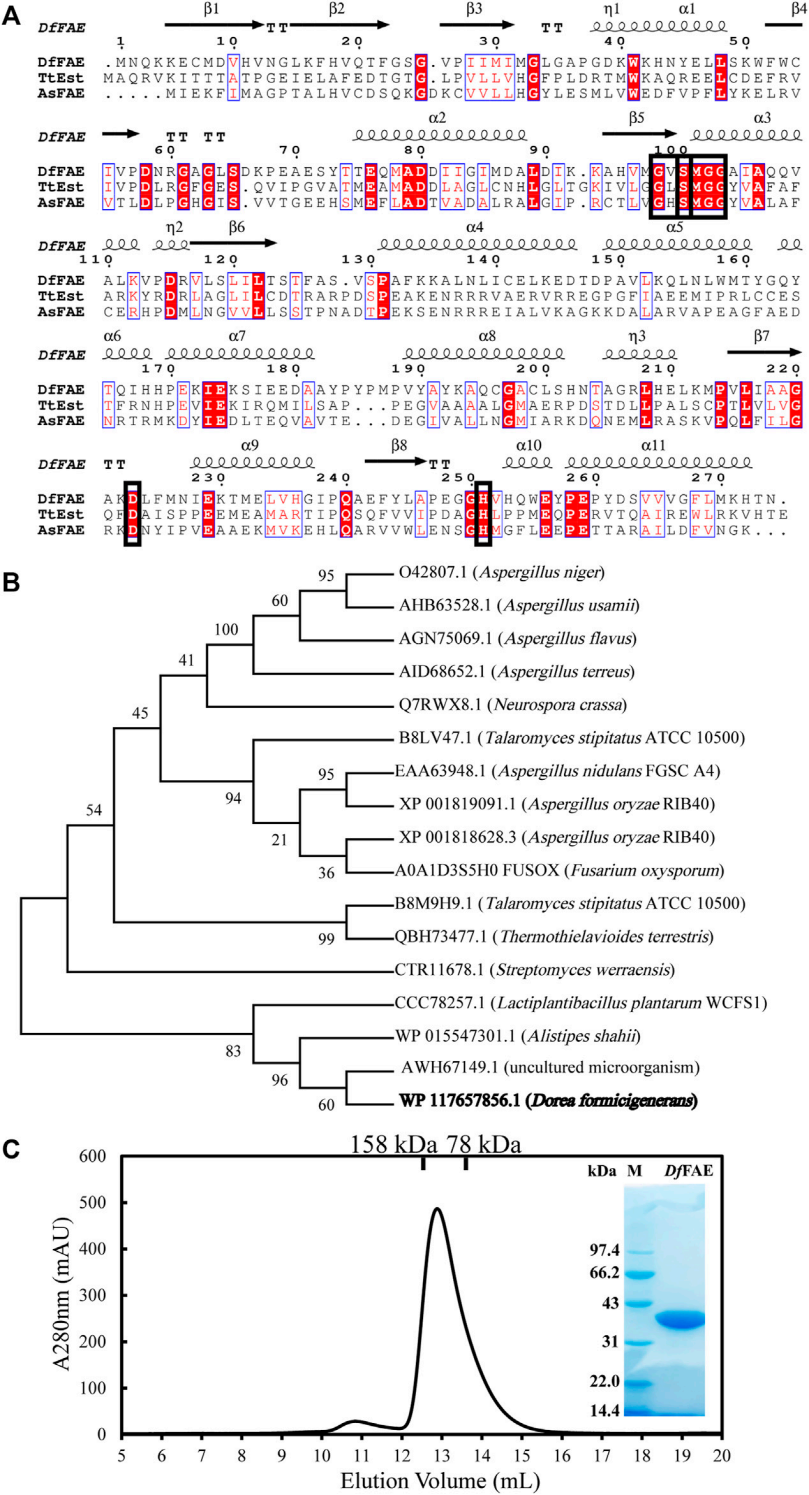
Sequence analysis and protein expression and purification of *Df*FAE. **(A)** Amino acid sequence alignment of *Df*FAE, *Ts*Est and *As*FAE. The ClustalX 2.0 tool was used to align the sequences, and the ESpript 3.0 program was used to display them. The alpha helices and beta strands are displayed above the sequences denoted as α and β, respectively. The red background denotes highly conserved residues. The black box denotes the highly conserved catalytic triad and pentapeptide GXSXG motif **(B)** Phylogenetic tree analysis of *Df*FAE and other classified FAEs. The sequences in the phylogenetic tree are, from top to bottom, *An*FAEA (O42807.1) of *Aspergillus niger*; *Au*FaeA (AHB63528.1) of *Aspergillus usamii*; *Af*FaeA (AGN75069.1) of *Aspergillus flavus*; *At*FaeA (AID68652.1) of *Aspergillus terreus*; *Nc*FaeD-3.544 (Q7RWX8.1) of *Neurospora crassa*; *Ts*FaeC (B8LV47.1) of *Talaromyces stipitatus*; *AN*1772.2 (EAA63948.1) of *Aspergillus nidulans*; *Ao*FaeB (XP_001818628.3) and *Ao*FaeC (XP_001819091.1) of *Aspergillus oryzae*; *Fo*FaeC (A0A1D3S5H0_FUSOX) of *Fusarium oxysporum*; *Sw*FAED (CTR11678.1) of *Streptomyces werraensis*; *LP*_0796 (CCC78257.1) of *Lactobacillus plantarum*; *As*FAE (WP_015547301.1) of *Alistipes Shahii*; FAE-Xuan (AWH67149.1) of *Soil Metagenomic Library*; *Df*FAE is shown in bold **(C)** SEC and SDS-PAGE analysis of the purified *Df*FAE. The elution volume is 12.9 ml, and the molecular mass markers are indicated above: 75 kDa standard protein corresponds to conalbumin, and 158 kDa standard protein corresponds to aldolase. The SDS-PAGE gel was stained with Coomassie blue. M represents the molecular weight marker of protein standards.

The full-length nucleotide sequence of *Df*fae is 825 bp and composed of 274 amino acids, which was defined as the α/β hydrolase in the NCBI protein database (accession number: WP_117657856.1). The theoretical molecular weight and isoelectric point were estimated as 30.5 kDa and 5.82, respectively, according to Expasy website (https://web.expasy.org/protparam/). The SignalP (Almagro Armenteros et al., 2019) analysis indicated that *Df*FAE lacks the possible signal peptide and thus might be a cytoplasmic protein. A BlastP search against the Protein Data Bank (PDB) database showed that *Df*FAE had the highest sequence homology of 29.66% with a thermophilic esterase *Tt*Est from *Thermogutta terrifontis* (PDB code: 4UHD) (Figure 1A). 16 characterized FAE sequences that were classified as A, B, C and D subtypes were used for phylogenetic tree analysis with *Df*FAE, containing both eukaryotic and prokaryotic origins. The results showed that these sequences were not clustered strictly in accordance with the FAE classification, and *Df*FAE is adjacent to type A FAEs of prokaryotic origin (Figure 1B). Sequence alignment of *Df*FAE with *Tt*Est and *As*FAE (sequence identity of 19.5%) identified three amino acids (Ser100, Asp223, and His251) as the putative catalytic triad of *Df*FAE. Ser100 is situated in the pentapeptide GXSXG consensus motif, which is a typical feature of esterases (Figure 1A).

To confirm the putative function, the recombinant *Df*FAE was overexpressed in *E. coli* T7 host cells and purified using Ni^2+^-NTA affinity column, followed by Source 15Q ion exchange and Superdex 200 gel filtration chromatographies. An obvious band at 34 kDa can be seen with 12% SDS-PAGE analysis, consistent with the theoretical molecular weight of *Df*FAE plus the N-terminal His_6_-tag, thrombin cutting site and T7-tag sequences from pET-28a (+) vector (34.1 kDa). The size exclusion chromatography (SEC) analysis showed that the apparent MW of the protein in solution was about 117 kDa, suggesting that the recombinant *Df*FAE is primarily trimeric and purified to homogeneity (Figure 1C).

**FIGURE 2:**
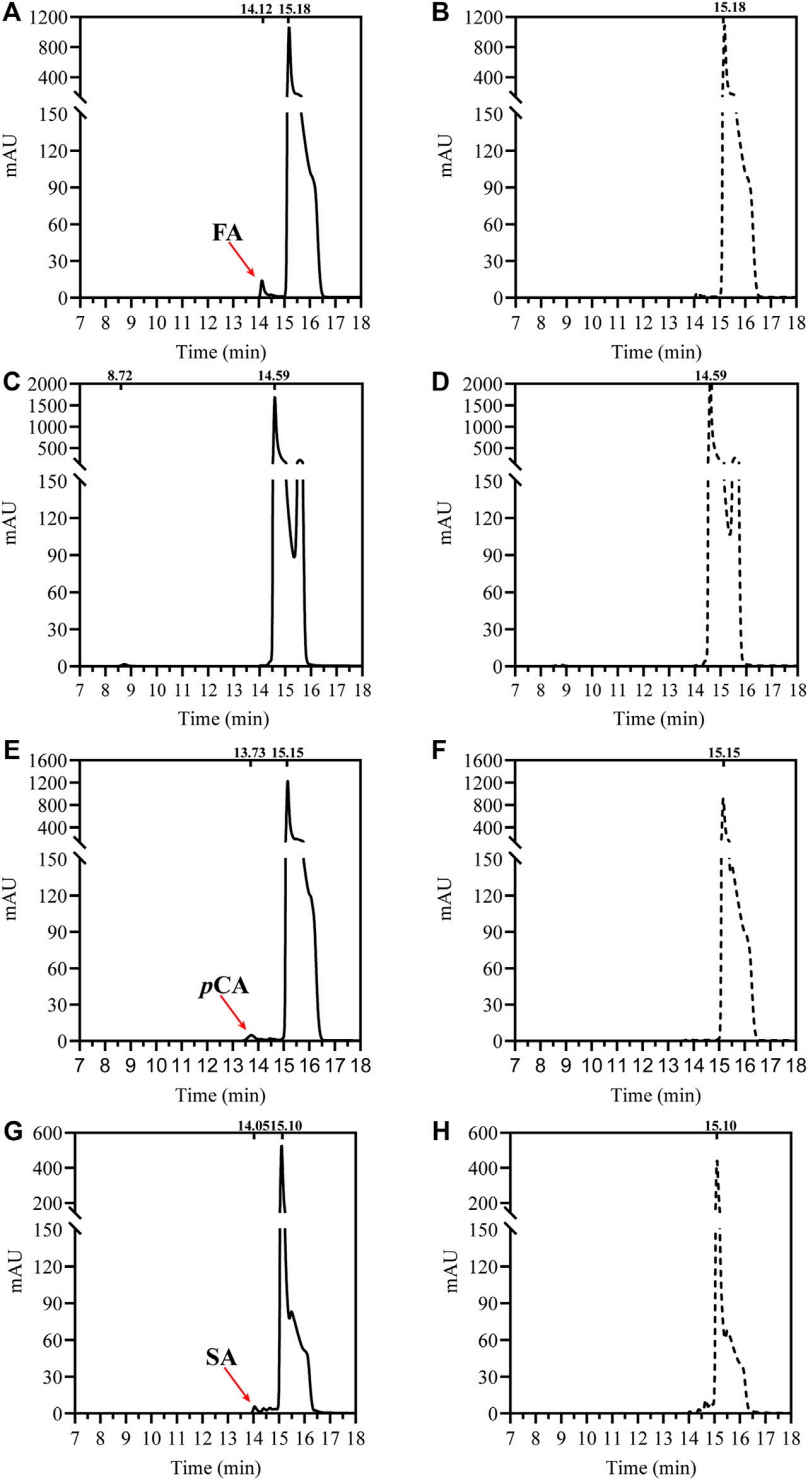
HPLC analysis of *Df*FAE-catalyzed hydrolysis of four model substrates of FAEs. **(A)** The product profile of *Df*FAE-catalyzed hydrolysis of MFA; **(B)** the blank control group of MFA without adding *Df*FAE; **(C)** The product profile of *Df*FAE-catalyzed hydrolysis of MCA; **(D)** the blank control group of MCA without adding *Df*FAE; **(E)** The product profile of *Df*FAE-catalyzed hydrolysis of M*p*CA; **(F)** the blank control group of M*p*CA without adding *Df*FAE; **(G)** The product profile of *Df*FAE-catalyzed hydrolysis of MSA; **(H)** the blank control group of MSA without adding *Df*FAE. The purified *Df*FAE (8 μM) was incubated with 1.25 mM substrates in 10 mM Tris-HCl buffer (pH 8.4) at 40 °C for 1 h. The positions of hydroxycinnamic acids and their esters detected in the assays are also indicated. The chromatograms were recorded at 323 nm.

To verify its feruloyl esterase activity, *Df*FAE was assayed using four model hydroxycinnamoyl ester substrates, MFA, MSA, MCA and M*p*CA. As shown in Figure 2, *Df*FAE can partially hydrolyze MFA, MSA and M*p*CA. However no detectable activity toward MCA was observed using the same enzyme load (a final concentration of 8 μM), indicating *Df*FAE can be classified as a type-A FAE.

### Biochemical Characterization and Substrate Specificity of *Df*FAE

**FIGURE 3:**
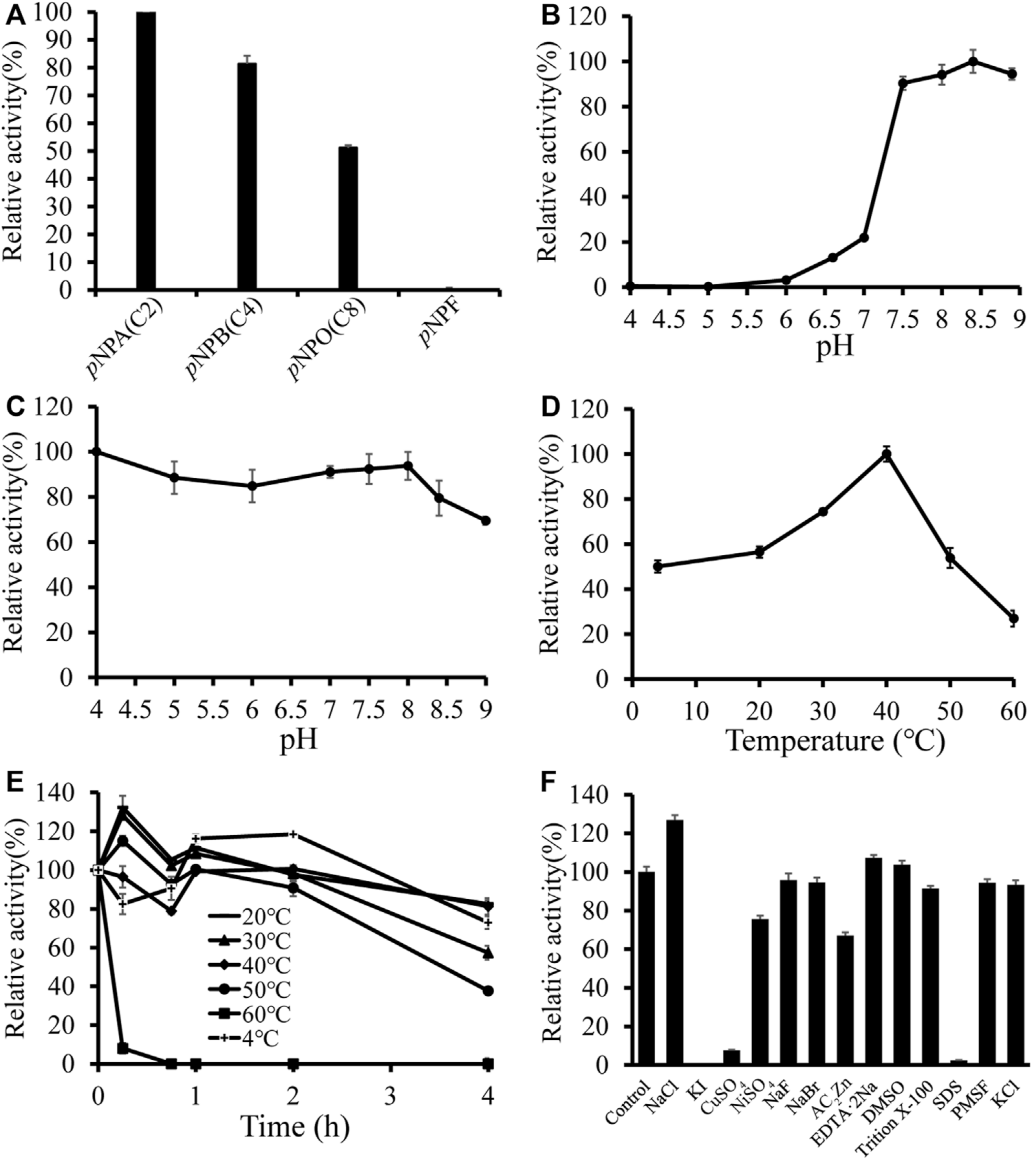
Biochemical characterization of *Df*FAE. **(A)** Substrate preference of *Df*FAE was measured with *p*-nitrophenyl esters. The observed maximum activity was defined as 100% **(B–C)** Effect of pH on *Df*FAE activity **(B)** and stability **(C)**. **(D–E)** Effect of temperature on *Df*FAE activity **(D)** and stability **(E)**. 4 °C, plus sign (+); 20 °C, minus sign (-); 30 °C, solid triangle (▲); 40 °C, solid tilted square (◆); 50 °C, solid circle (●); 60 °C, solid box (■). The activity measured without preincubation is defined as 100% **(F)** Effect of various metal ions and detergents on *Df*FAE activity. The activity of the blank control group was taken as 100%. The error bars indicated the standard deviations obtained from three independent experiments.

To further analyze the substrate preference of *Df*FAE, the enzymatic activity was measured spectrophotometrically by hydrolysis of four *p*-nitrophenyl esters with different chain lengths, including *p*NPA (C2), *p*NPB (C4), *p*NPO (C8) and *p*NPF as substrates. The results indicated that *Df*FAE was most active toward short-chain substrate *p*NPA, while its hydrolysis activity toward *p*NPB and *p*NPO decreased to 81 and 51%, respectively, compared with that of *p*NPA. Under the same experimental conditions, the hydrolysis of long-chain *p*NPF by *Df*FAE could not be detected, suggesting *Df*FAE had a substrate preference toward short-chain esters (Figure 3A).

Then the enzymatic characteristics of *Df*FAE were assayed using the more preferred substrate *p*NPA. *Df*FAE has an optimal pH of 8.4 and maintains higher activity at alkaline conditions, almost 90% of its maximum activity. The enzyme became almost inactive when the pH decreased below 6.0 (Figure 3B). The pH stability was assessed by incubating *Df*FAE at 4 °C in different pH buffers for 4 h before measured the activity in the optimum conditions. *Df*FAE was stable over a broad pH range from 4.0 to 8.0 with more than 80% residual activity maintained (Figure 3C). Furthermore, the esterase activity of *Df*FAE was tested over a temperature range of 4–60°C, showing that *Df*FAE exhibiting an optimum temperature of 40°C and remaining at 54 and 27% of its maximal activity at 50 and 60°C, respectively (Figure 3D). For thermal stability, *Df*FAE was stable below 50°C, while its activity drastically decreased after incubation at 60°C for 15 min (Figure 3E). At a final concentration of 1 mM (w/v) or 1% (v/v), the effect of metal ions and different detergents on *Df*FAE activity was also investigated (Figure 3F). It can be observed that the addition of KI completely inactivated *Df*FAE, while CuSO_4_ and SDS significantly reduced the enzymatic activity to 8 and 2%, respectively. NiSO_4_ (76%) and ZnAC_2_ (67%) showed a mild inhibitory effect, while activity could be significantly promoted by 1 mM NaCl (127%). The other substances showed no significant effect on *Df*FAE enzymatic activity.

We further determined the kinetic properties of *Df*FAE toward *p*NPA, *p*NPB and *p*NPO. The results are summarized in Table 1. *Df*FAE showed a higher substrate preference for hydrolyzing *p*NPA on which it exhibited the highest catalytic efficiency with a *k*_cat_/*K*_m_ value of 7.73 s^−1^mM^−1^, almost 3.5- and 21.8-fold higher than that of *p*NPB (2.23 s^−1^mM^−1^) and *p*NPO (0.35 s^−1^mM^−1^), respectively. The hydrolytic activity of *Df*FAE toward *p*NPA is comparable or much higher than other gut-derived FAEs, including BiFae1A (*k*_cat_/*K*_m_ value of 11.9 s^−1^mM^−1^) (Wefers et al., 2017), BoCE1 (*k*_cat_/*K*_m_ value of 0.5 s^−1^mM^−1^) (Kmezik et al., 2020) and *As*FAE (*k*_cat_/*K*_m_ value of 0.84 s^−1^mM^−1^) (Wei et al., 2021). Nonetheless, *Df*FAE showed a slightly higher affinity toward *p*NPB (*K*_m_ value of 0.36 mM) compared with *p*NPA and *p*NPO (*K*_m_ values of 0.57 and 0.53 mM, respectively), indicating the three substrates are easily accessible to the active site region.

**TABLE 1:** Kinetic parameters of wild-type *Df*FAE and its mutants.

Substrate	Enzyme	*K*_m_ (mM)	*k*_cat_ (s^−1^)	*k*_cat_/*K*_m_ (s^−1^mM^−1^)
*p*NPA/*p*NPB/*p*NPO	S100A		N.D^a^	
	H251A			
	D223A			
*p*NPA	WT	0.572 ± 0.05657	4.423 ± 0.1647	7.733 ± 0.1361
	L34A	1.464 ± 0.122	3.579 ± 0.106	2.445 ± 0.1130
	A36D	9.574 ± 4.3	26.02 ± 7.897	2.718 ± 0.7526
	V99A	1.702 ± 0.2479	13.54 ± 0.9546	7.955 ± 0.2162
	K153A	0.9654 ± 0.08571	6.023 ± 0.1879	6.239 ± 0.12
	N156A	0.6447 ± 0.04059	14.79 ± 0.3327	22.941 ± 0.0855
	T160A	0.9086 ± 0.2637	8.608 ± 1.14	9.474 ± 0.4227
	V252A	0.2889 ± 0.0538	12.75 ± 0.5099	44.133 ± 0.2262
	W255A	0.8097 ± 0.1824	12.05 ± 0.8912	14.882 ± 0.2992
	P149A	0.8037 ± 0.1078	17.43 ± 0.8093	21.687 ± 0.1806
	D180A	0.5243 ± 0.07629	3.419 ± 0.159	6.521 ± 0.1920
	P186A	0.824 ± 0.2421	11.32 ± 1.207	13.738 ± 0.4004
*p*NPB	WT	0.3573 ± 0.06862	0.7978 ± 0.04357	2.233 ± 0.2467
	N156A	0.3343 ± 0.04369	2.85 ± 0.09965	8.525 ± 0.1657
	V252A	0.08361 ± 0.00865	1.073 ± 0.02156	12.833 ± 0.1235
	W255A	0.3104 ± 0.02622	1.196 ± 0.0264	3.853 ± 0.1065
	P149A	1.471 ± 0.2324	3.538 ± 0.2196	2.405 ± 0.2201
	P186A	0.7984 ± 0.08708	1.07 ± 0.04159	1.34 ± 0.1479
	K153A	1.407 ± 0.09988	1.224 ± 0.03517	0.87 ± 0.0997
*p*NPO	WT	0.5262 ± 0.07357	0.1864 ± 0.008647	0.354 ± 0.1862
	N156A	0.2821 ± 0.04357	0.2227 ± 0.008196	0.789 ± 0.1913
	V252A	0.09987 ± 0.01876	0.5198 ± 0.01795	5.205 ± 0.2224
	W255A	0.1782 ± 0.01366	2.87 ± 0.05431	16.105 ± 0.0956
	P149A	0.4288 ± 0.03531	1.076 ± 0.02427	2.509 ± 0.1049
	P186A	0.5728 ± 0.05851	0.2177 ± 0.006844	0.38 ± 0.1336
	K153A	0.7307 ± 0.08584	0.1339 ± 0.005545	0.183 ± 0.1589

a N.D. not detectable.

### Structure Prediction of *Df*FAE by AlphaFold2

**FIGURE 4:**
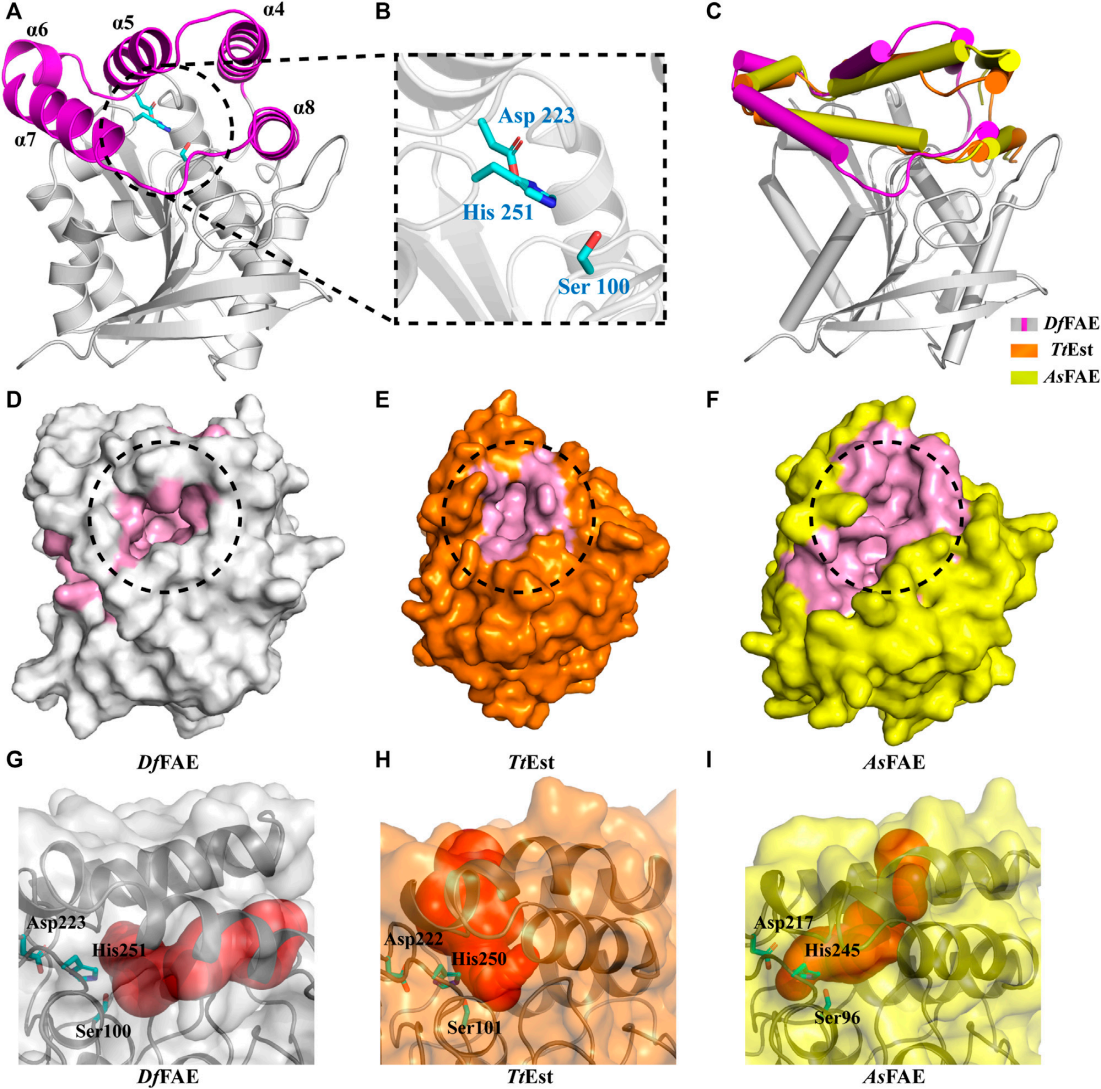
**(A)** Overall structure of *Df*FAE. The magenta and gray colors represent the core and cap domains, respectively. **(B)** Close-up views of the catalytic triad. Residues in the catalytic triad are represented as cyan sticks **(C)** Comparison of the cap domain of *Df*FAE (magenta and gray), *Tt*Est (orange) and *As*FAE (yellow). **(D–E)** Surface representation of the active site pocket of *Df*FAE (D, gray), *Tt*Est (E, orange) and *As*FAE (F, yellow). The active site pocket region is marked in pink, and the entrance is highlighted by black dotted circle **(G–I)** *Df*FAE, *Tt*Est and *As*FAE substrate access tunnel conformations calculated by CAEVER 3.0, shown as red spheres of different radius.

To further illustrate the structural basis for the catalytic properties of *Df*FAE, we attempted to crystallize it but unfortunately failed after multiple rounds of trying. Therefore, we used AlphaFold2, a newly introduced structure prediction program with unprecedented accuracy, to build the 3D-structure of *Df*FAE based on its amino acid sequence. The pLDDT values under the best model were greater than 80 for 98% of the residues and less than 50 for only the top two residues, indicating that the model was generally plausible. The overall structure of *Df*FAE contains a typical α/β hydrolase fold core domain composed of a central eight-stranded β-sheet flanked by six helices, and a cap domain formed by α4-α8 helices positioned atop the core domain (Figure 4A). The active site region responsible for substrate binding and catalysis is located between the core and cap domains. The entrance of the active site pocket is formed by two flexible surface loops between α4-α5 (Asp146-Pro149) and α7-α8 (Ala182-Val189) from the cap domain. The catalytic Ser100 is located at the nucleophilic elbow between the β5 sheet and the α3 helix, lying within the hydrogen-bond distance (4.7 Å) with the general base His251 which further interacts with Asp223 to form a charge relay system (Figure 4B). The entire catalytic triad is encapsulated inside the active site pocket and less exposed to solvent. Leu34 and Met101 potentially contribute to the formation of the oxyanion hole according to the sequence alignment (Figure 1A).

The cap domain is highly variable across different esterase structures and affects the conformation of the active site pocket (Goldstone et al., 2010; Bains et al., 2011; Wefers et al., 2017). Structural superimposition of *Df*FAE with homologous *Tt*Est and *As*FAE revealed considerable variation in the orientation of the cap domain, leading to a significant difference in the size of active site pocket and the position of its entrance (Figures 4C–F). Notably, *Df*FAE features a relatively close and deep pocket in which the entrance is partially covered by the connecting loop between α7 and α8, three residues longer than that in *Tt*Est and *As*FAE (Figure 1A; Supplementary Figure S1). The DoGSiteScorer analysis (Volkamer et al., 2010; Volkamer et al., 2012) of the size of active site pocket indicated that *Df*FAE has the largest pocket with the volume of 761.92 Å^3^, while the volumes of *As*FAE and *Tt*Est active site pocket are calculated as 701.82 Å^3^ and 514.05 Å^3^, respectively. These observations suggest *Df*FAE possess an active site pocket with narrow entrance but large internal cavity to accommodate long-chain substrates, and may explain why *Df*FAE has the ability to catalyze the hydrolysis of *p*NPO, while the other two esterases do not (Sayer et al., 2015; Wei et al., 2021).

To more intuitively illustrate the conformational differences of the active site pocket of *Df*FAE and its homologous proteins, the potential substrate access tunnel which refers to a pathway connecting the protein outer surface toward the buried catalytic center was identified using CAVER 3.0 (Chovancova et al., 2012; Kokkonen et al., 2019). The substrate access tunnel of *Df*FAE is formed at the interface of a four-helix bundle composed of α4, α5, α7 and α8 helices from the cap domain with a length of 22 Å and an average radius of 2.63 Å (Figures 4G; Supplementary Figures S2A). In contrast, the tunnel entrance of *Tt*Est and *As*FAE is positioned in the middle of the top two helices (α4-α5) from the cap domain, and the overall tunnel is nearly vertical to that of *Df*FAE (Figures 4H, I; Supplementary Figures S2B, C). These results indicated that the three structurally similar homologous esterases have totally different active site topology.

### Selection of Mutagenesis for Enhanced Activity

**FIGURE 5:**
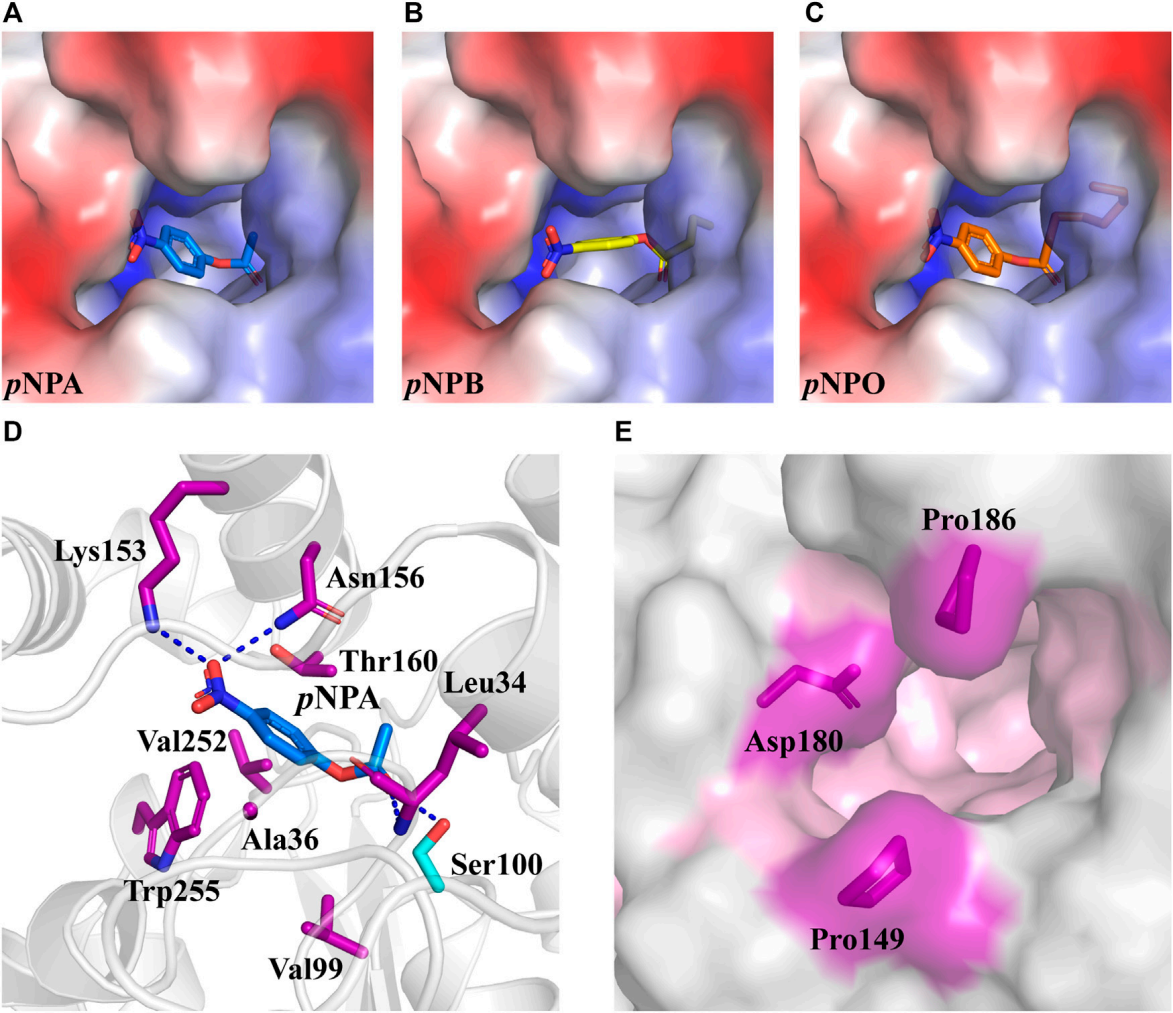
The optimal conformation of *p*NPA, *p*NPB and *p*NPO docking in *Df*FAE. (A–C) *Df*FAE is shown as surface representation, colored by electrostatic potentials (positive, blue; negative, red). *p*NPA (A, blue), *p*NPB (B, yellow) and *p*NPO (C, orange) are shown as sticks. **(D)** Cartoon representation of *p*NPA binding to the active site of *Df*FAE. The catalytic Ser100 is shown as a cyan stick, and other interacting residues are shown as purple sticks. Hydrogen bonding forces are shown as blue dashed lines **(E)** Surface representation of mutation sites at the entrance of binding pocket. The binding pocket area colored in pink, mutation site residues are shown as purple sticks.

Rational design provides an effective tool to modulate the biocatalytic properties of enzymes. In previous studies, rational design strategies have been successfully applied to several FAEs with improved enzymatic activity or thermostability by modifying the active site (Antonopoulou et al., 2018; Liu et al., 2021; Yang et al., 2022). *Df*FAE features a narrow active site pocket including a substrate access tunnel deeply into the protein, which may reduce the entry of the large carboxyl portion of the ester substrate to the catalytic residues. Hence this study aims to engineer the active site for enhanced catalytic efficiency of *Df*FAE. In order to identify the residues that likely contribute to substrate binding, the predicted *Df*FAE structure was subjected to molecular docking by Autodock_vina tool (Trott and Olson, 2010) with *p*NPA, *p*NPB and *p*NPO. All three *p*NP-esters showed the similar mode of binding into the active site pocket (Figures 5A–C). The distance between the catalytic Ser100 and the carbonyl carbon atom of each substrate is approximately 3.6 Å. In the docking conformation of *p*NPA that is most preferred by *Df*FAE, the nitro group oxygen makes a salt bridge to Lys153 and also a hydrogen bond to Asn156 on helix α5 from cap domain, while the acyl group oxygen is stabilized by hydrogen bonds to Leu34 main chain and Ser100 side chain in line for catalysis. Residues within 5 Å of *p*NPA (Ala36, Val99, Val252 and Trp255 from the core domain and Thr160 from the cap domain) are expected to form hydrophobic interactions (Figure 5D). All these residues are not conserved according to sequence alignment (Figure 1A) and were selected for mutagenesis. In addition, Pro149, Asp180 and Pro186 from the cap domain occupied the entrance to the substrate access tunnel and were mutated to Ala in an aim to open up the catalytic pocket (Figure 5E).

### Kinetic Analysis of *Df*FAE Mutants

**FIGURE 6:**
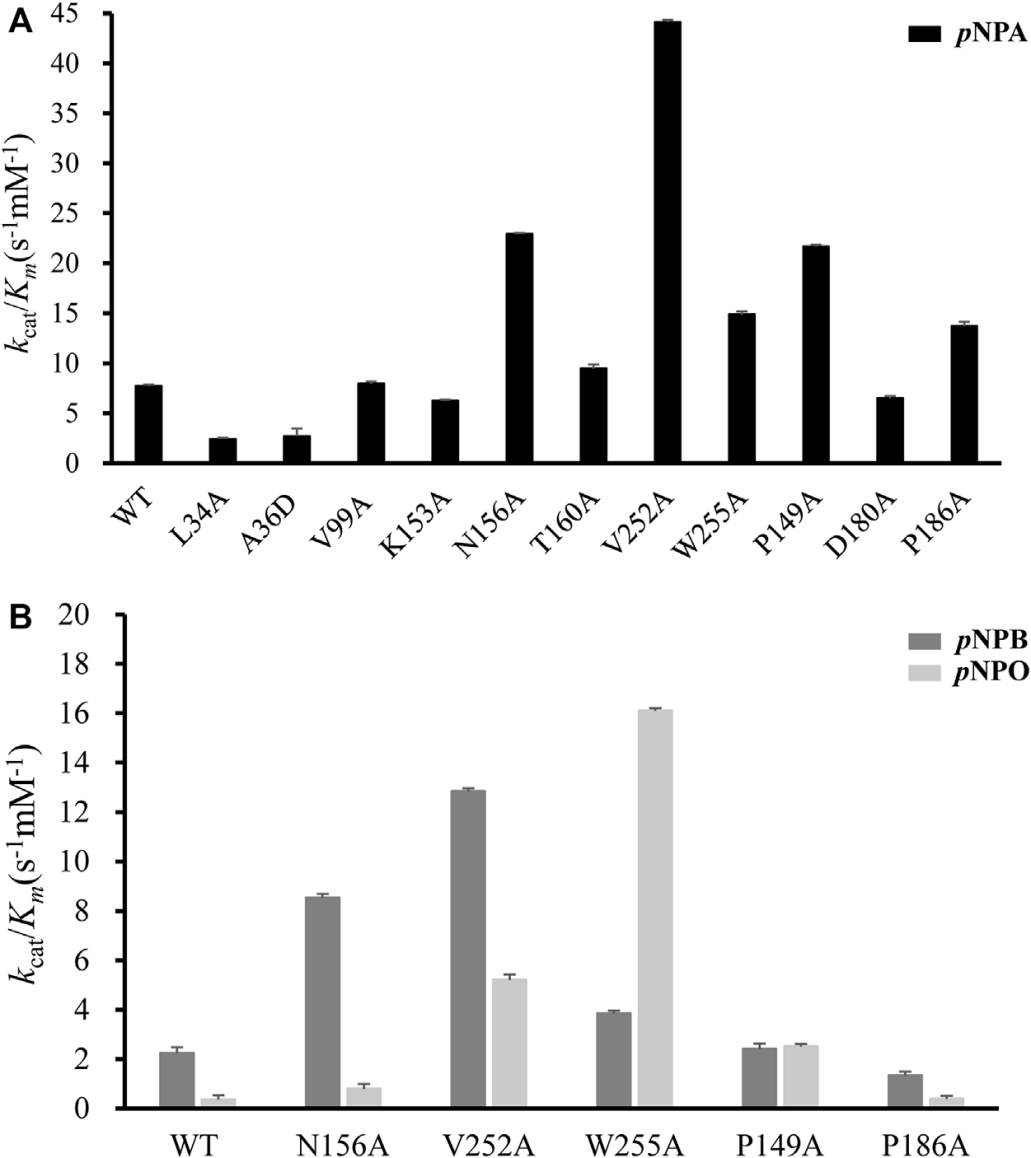
Comparison of the catalytic efficiency (represented by *k*_cat_/*K*_m_) measured for wild-type *Df*FAE and its mutants. **(A)** The *k*_cat_/*K*_m_ values on *p*NPA for WT and all mutants **(B)** The *k*_cat_/*K*_m_ values of WT and some mutants on *p*NPB and *p*NPO. The error bars indicated the standard errors from three independent experiments.

All mutants were overexpressed in *E. coli* T7 and purified by Ni^2+^-NTA and ion exchange chromatographies. Then kinetic experiments were performed to evaluate the role of each residue (Figure 6A; Table 1). Mutation of the catalytic triad Ser100, His251 and Asp223 resulted in the complete elimination of enzymatic activity, confirming their essential roles in acyl ester hydrolysis. In the case of residues involved in substrate recognition, V252A displayed the highest catalytic efficiency toward *p*NPA with both decreased *K*_m_ and increased *k*_cat_, which was 5.71-fold higher (*k*_cat_ */K*_m_ value of 44.13 s^−1^mM^−1^) than the wild type (*k*_cat_ */K*_m_ value of 7.73 s^−1^mM^−1^). Similarly, N156A and W255A showed a slightly decreased *K*_m_ but an elevated *k*_cat_ values, resulting in enhanced catalytic efficiency compared to the wild type (*k*_cat_ */K*_m_ value of 22.94 s^−1^mM^−1^ and 14.88 s^−1^mM^−1^, respectively). These results suggested that Ala replacement of residues with bulky side chain possibly leads to relieving the steric hindrance and increasing the internal space of active site pocket which could provide additional degree of freedom for substrate positioning and accelerate substrate turnover (*k*_cat_). In contrast, Ala36 mutated to a large Asp showed a *K*_m_ value increased to 16.74-fold but a *k*_cat_ */K*_m_ value decreased to 0.35-fold. Leu34 is a likely candidate to stabilize the oxyanion hole and its mutation reduced the enzymatic activity remarkably. V99A, T160A and K153A showed little effects on *Df*FAE catalytic efficiency but an apparent decrease in substrate binding affinity, suggesting the three residues may play a role in the substrate binding and catalysis process. Alternatively, in the case of amino acids involved in substrate entry, the catalytic efficiency of P149A and P186A for *p*NPA hydrolysis increased by about 3-fold and 2-fold, respectively, while D180A showed a negligible effect on *Df*FAE activity, indicating that Pro149 and Pro186 play an essential role in gating the substrate access tunnel.

**FIGURE 7:**
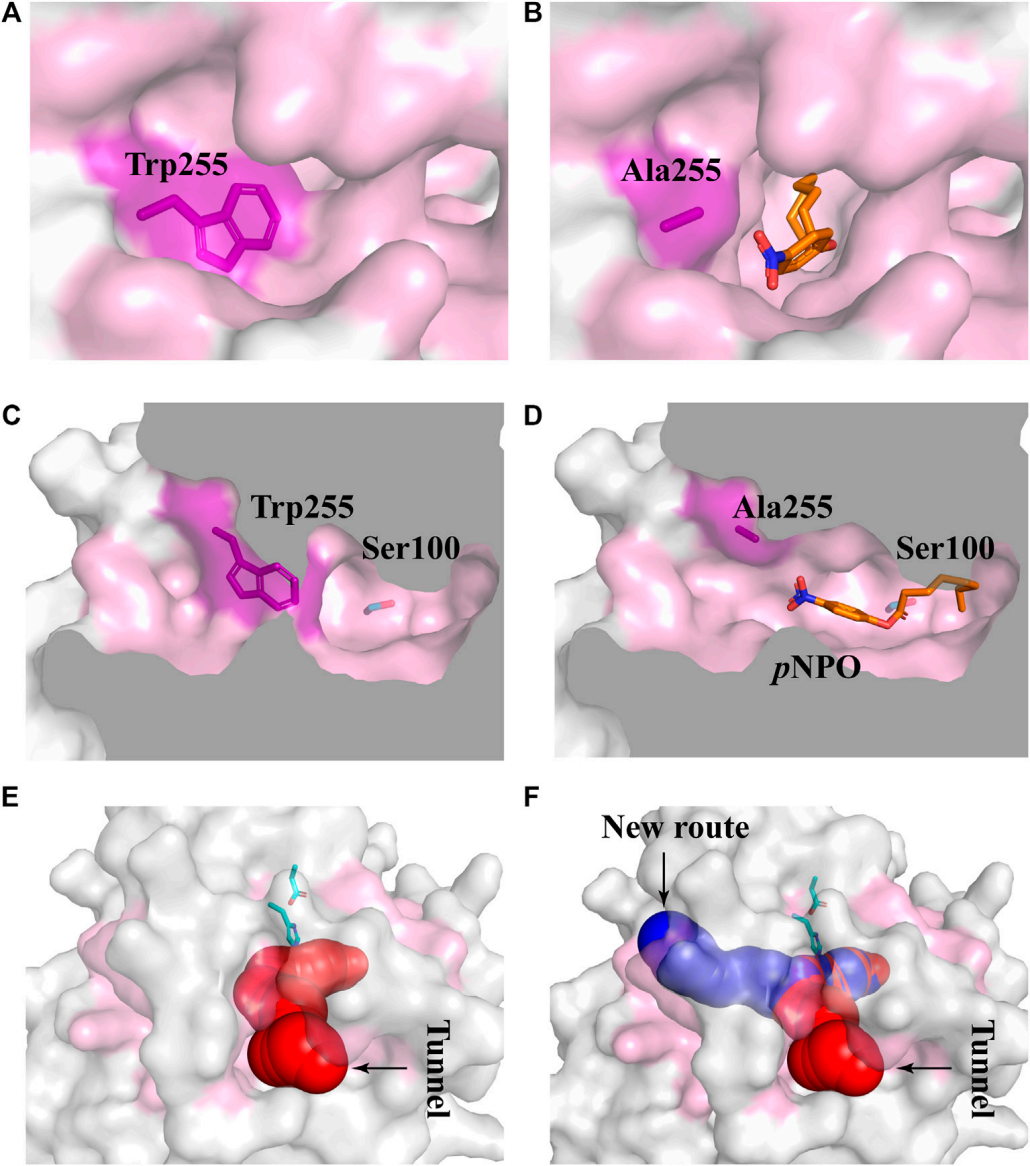
Structural differences in the substrate access tunnel of wild-type *Df*FAE and W255A mutant. **(A–B)** The entrance of the new route of W255A **(B)** and the main substrate access tunnel of WT **(A)**. **(C–D)** Cutaway view of the new route of W255A **(D)** and the main substrate access tunnel of WT **(C)**. **(E–F)** Comparison of the substrate access tunnel conformation of W255A **(F)** calculated by CAVER 3.0 and that of WT **(E)**. The binding pocket is colored by pink. The ligand *p*NPO is shown as orange stick, the new route and the main substrate access tunnel are shown as blue and red, respectively.

Subsequently, five mutants V252A, N156A, P149A, P186A, and W255A which had higher activities toward *p*NPA were selected to further determine the kinetic parameters toward *p*NPB and *p*NPO substrates. In particular, the *k*_cat_ */K*_m_ values of V252A were respectively 5.75- and 14.7-fold higher toward *p*NPB and *p*NPO than that of the wild-type, and the *k*_cat_ */K*_m_ values of N156A toward *p*NPB and *p*NPO enhanced 3.69- and 2.23-fold, respectively, compared with the wild-type. On the other hand, mutation of Pro149 resulted in 7.09-fold higher catalytic efficiency toward *p*NPO but have a minor influence on *p*NPB, while P186A performed poorly on both substrates. Interestingly, we observed W255A gained a higher preference toward *p*NPO with 45.5-fold increase in catalytic efficiency (*k*_cat_ */K*_m_ value of 16.11 s^−1^mM^−1^) compared with the wild-type, while its activities toward *p*NPA and *p*NPB were 1.92- and 1.73-fold, respectively. To determine the mechanisms underlying the influences of W255A on *Df*FAE activity, we reanalyzed the structure of active site pocket in *Df*FAE and observed that besides the main substrate access tunnel there was still a side part in the internal space that is partitioned by the bulky side chain of Trp255 (Supplementary Figure S3; Figures 7A, C). Substitution of Trp255 to small Ala broadened internal cavity and possibly provided a new route for long-chain substrate easy access to the catalytic center (Figures 7B, D). The binding energy of *p*NPO in the new route is -7.0 kcal/mol, the same as the main substrate access tunnel (-7.0 kcal/mol). Therefore, these results indicated that modification of residues inside tunnel (Val252, Asn156 and Trp255) play a more vital role in improving *Df*FAE activity and broadening substrate profile for ester hydrolysis compared with residues gating the tunnel (Pro149, Asp180 and Pro186).

## Conclusion

In conclusion, this study characterized a novel type-A FAE from gut-derived bacteria *D. formicigenerans* (*Df*FAE). *Df*FAE showed a higher preference for short-chain esters, possibly due to its narrow and relatively closed active site pocket, including a deep substrate access tunnel. Structure-guided mutagenesis within the active site identified the mutants inside the cavity play a more vital role in enhancing catalytic efficiency toward *p*NP-esters by expanding the internal space of the tunnel. Moreover, W255A created a possible new route for substrate entry and showed a higher preference toward long-chain *p*NPO. Collectively, these results revealed the molecular determinant for how the substrate preference of *Df*FAE is influenced by its active site architecture and the applicability of the rational design strategy for obtaining enhanced biocatalysts, which can be referenced by other FAEs and gut-derived enzymes.

## Data Availability

The original contributions presented in the study are included in the article/Supplementary Material further inquiries can be directed to the corresponding authors.
