# Supplementary material for: Engineering the Active Site Pocket to Enhance the Catalytic Efficiency of a Novel Feruloyl Esterase Derived From Human Intestinal Bacteria Dorea formicigenerans
Source: Front Bioeng Biotechnol. 2022 Jun 20;10:936914. doi: 10.3389/fbioe.2022.936914 (PMC9251316; doi:10.3389/fbioe.2022.936914)
Supplement: Supplementary file 1 [file Table1.DOCX]

Supplementary Material

# Supplementary Figures


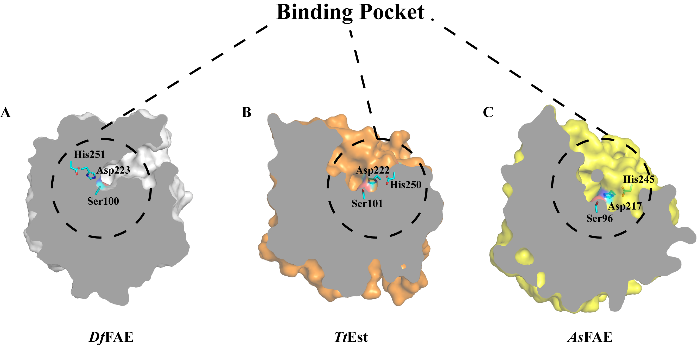


**Supplementary Figure 1.** Cutaway view of the substrate binding pocket conformation of (A) *Df*FAE (in gray), (B) *Tt*Est (in orange), (C) *As*FAE (in yellow) are highlighted in black dotted circle. Residues of the catalytic triad are shown as cyan sticks.


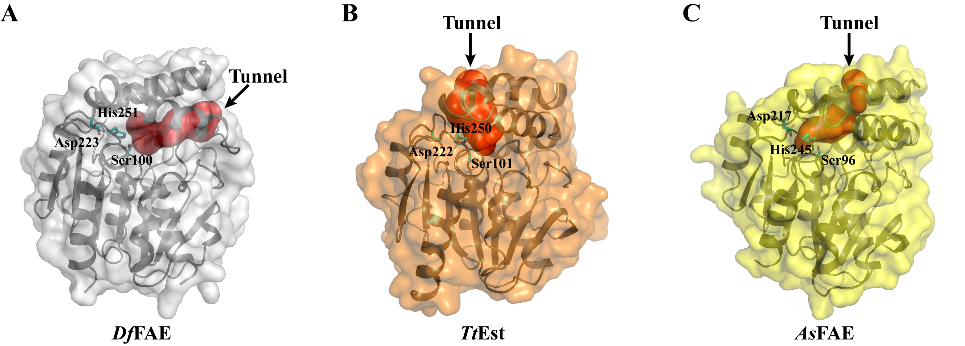


**Supplementary Figure 2.** (A) *Df*FAE (in gray), (B) *Tt*Est (in orange), (C) *As*FAE (in yellow), catalytic tunnel conformations calculated by CAEVER 3.0, shown as red spheres of different radius. The catalytic triad residues are shown as cyan sticks. Proteins are displayed as surface and cartoon.


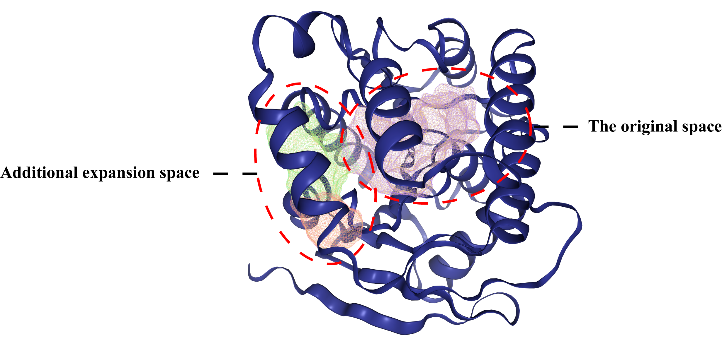


**Supplementary Figure 3.** Protein*p*lus website online tool DoGSiteScorer for *Df*FAE substrate binding pocket volume calculation and partition. The original space colored by purple, the additional expansion space colored by green and orange.
