# Supplementary figures and images for: Engineering the Active Site Pocket to Enhance the Catalytic Efficiency of a Novel Feruloyl Esterase Derived From Human Intestinal Bacteria Dorea formicigenerans
Source: Front Bioeng Biotechnol. 2022 Jun 20;10:936914. doi: 10.3389/fbioe.2022.936914 (PMC9251316; doi:10.3389/fbioe.2022.936914)

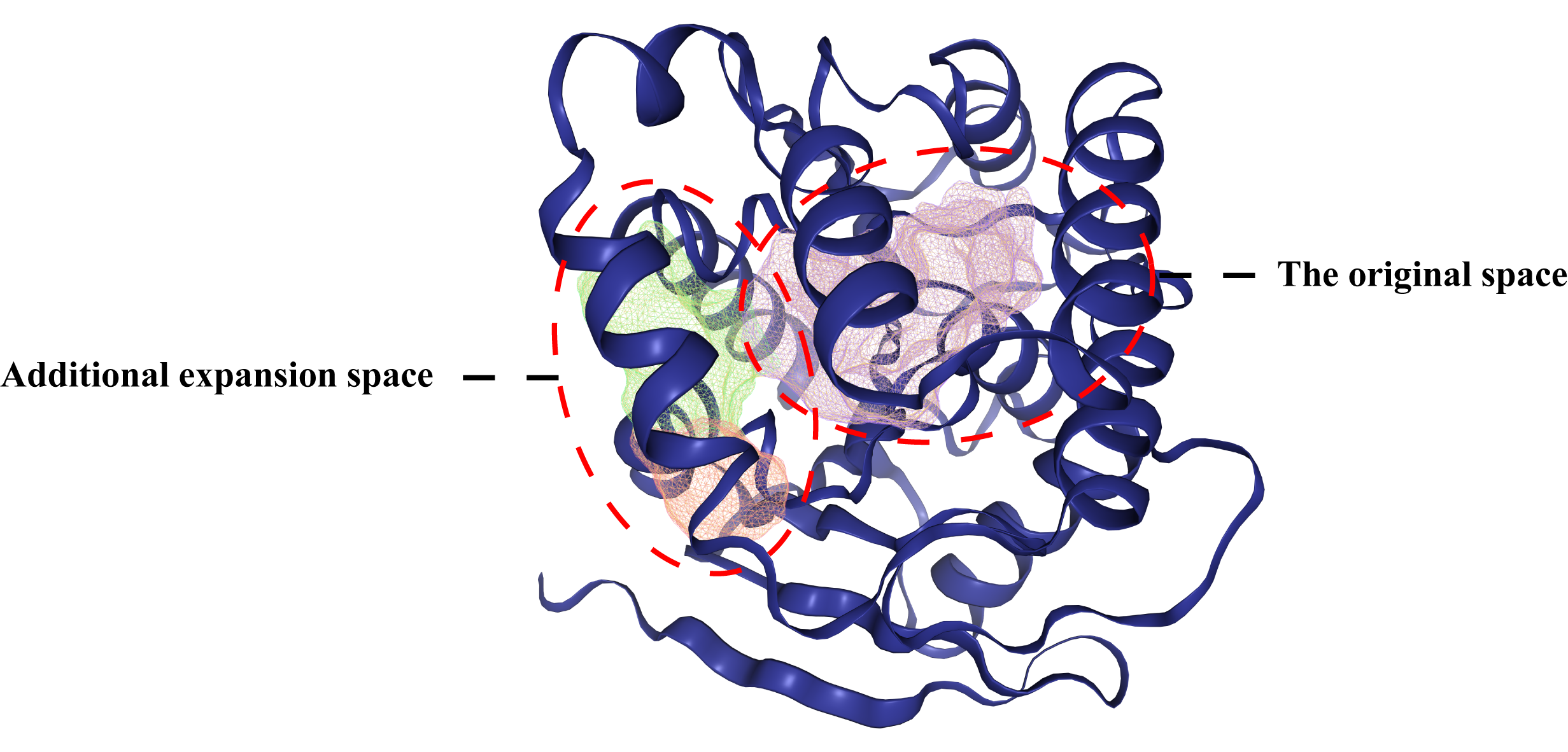

Supplement: Supplementary file 2 [file Image3.TIF]

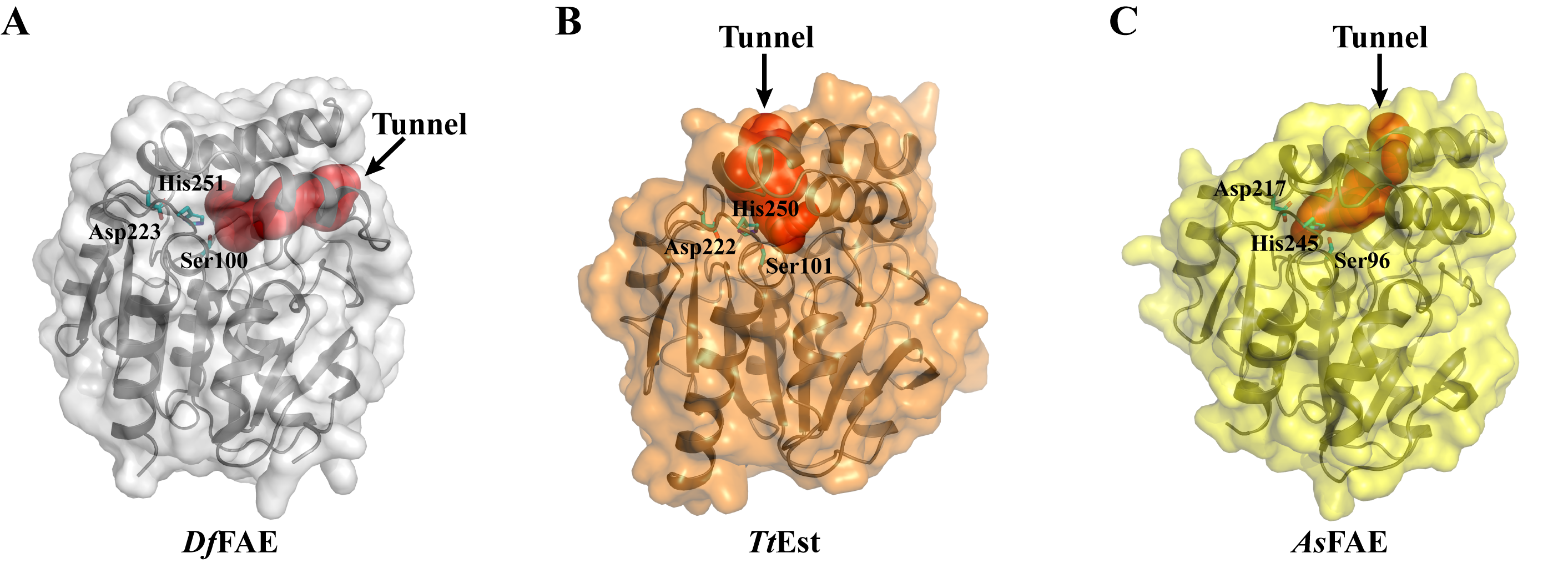

Supplement: Supplementary file 3 [file Image2.TIF]

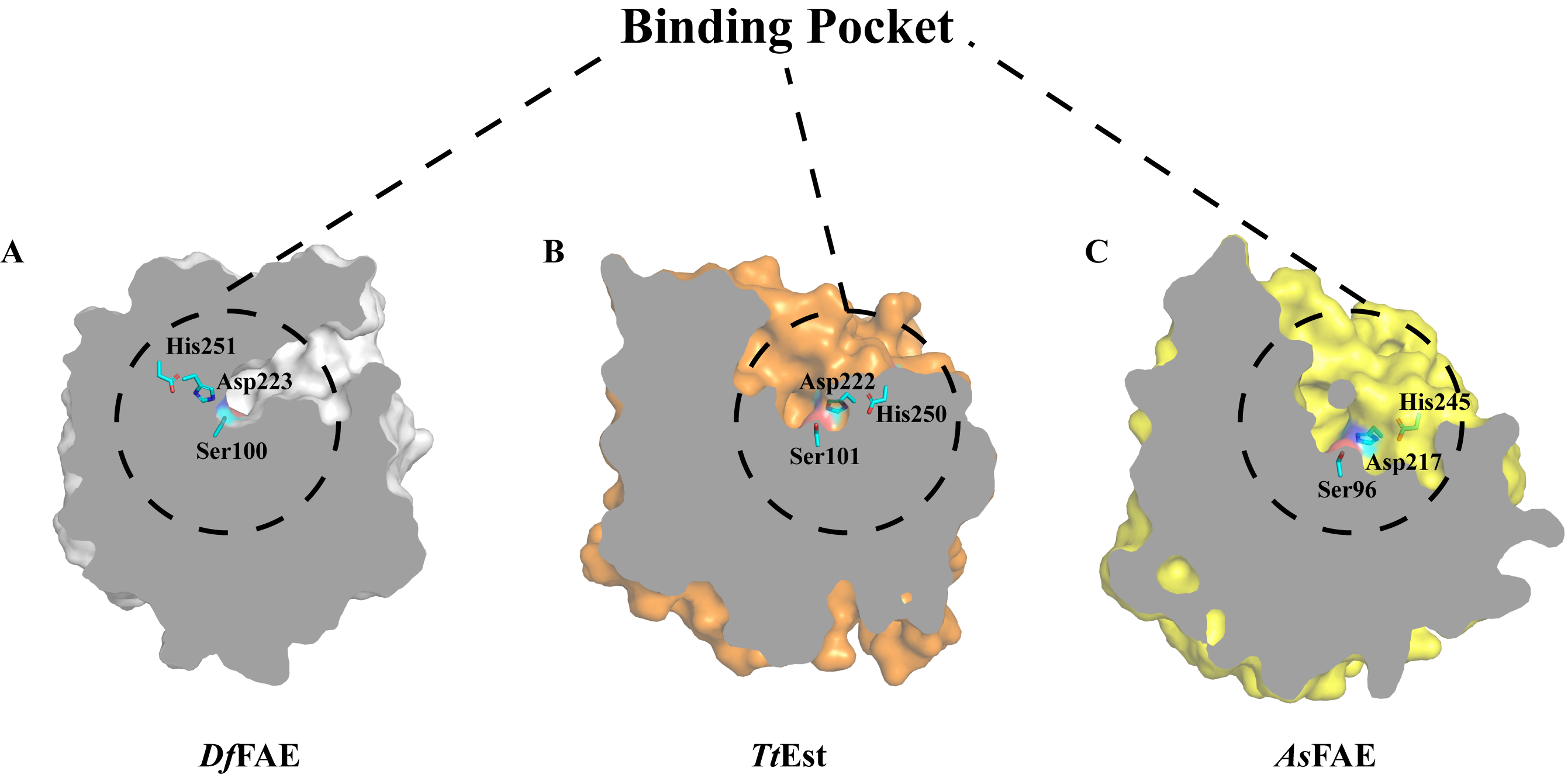

Supplement: Supplementary file 4 [file Image1.TIF]
